# Supplementary material for: Upconversion nanocomposite for programming combination cancer therapy by precise control of microscopic temperature
Source: Nat Commun. 2018 Jun 5;9:2176. doi: 10.1038/s41467-018-04571-4 (PMC5988832; doi:10.1038/s41467-018-04571-4)
Supplement: Supplementary file 1 — Supplementary Information [file 41467_2018_4571_MOESM1_ESM.pdf]

# Supplementary Information

Zhu et al. Upconversion Nanocomposite for Programming Combination Cancer Therapy by Precise Control of Microscopic Temperature

# Upconversion Nanocomposite for Programming Combination Cancer Therapy by Precise Control of Microscopic Temperature

Xingjun Zhu,† Jiachang Li,† Xiaochen Qiu,† Yi Liu,† Wei Feng,†* Fuyou Li†*

† Department of Chemistry & Institutes of Biomedical Sciences & State Key Laboratory of Molecular Engineering of Polymers, Fudan University, 220 Handan Road, Shanghai 200433, P.R. China

Correspondence should be addressed to W.F. (fengweifd@fudan.edu.cn) and F.Y.L. (fyli@fudan.edu.cn).

## Supplementary Figures

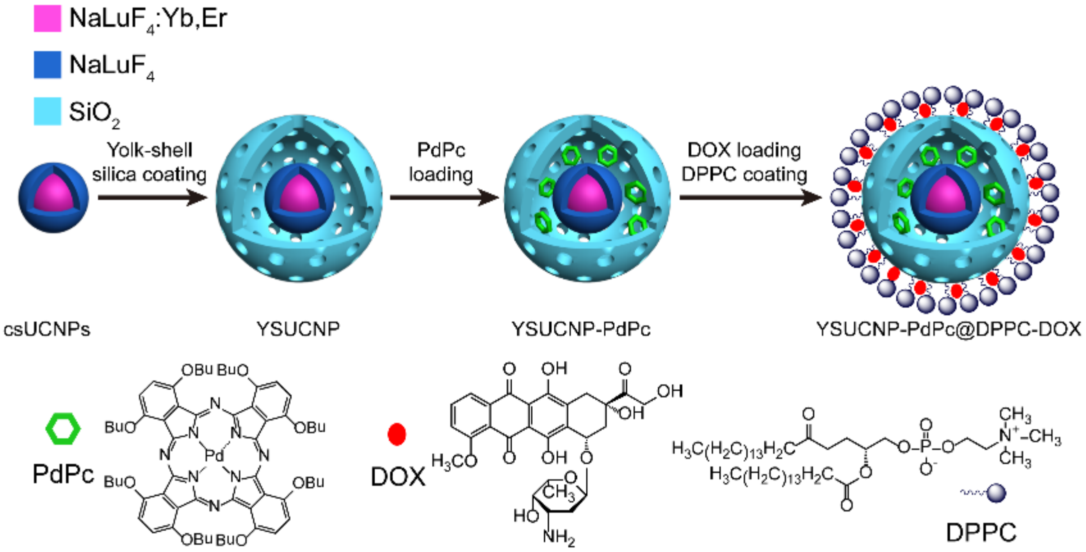


**Supplementary Figure 1.** Schematic diagram of the synthetic routine of TR-UCNS. Upconversion emissive core of $NaLuF_4$:Yb,Er@$NaLuF_4$ (csUCNPs) were first synthesized by the solvothermal method. Then, a yolk-shell like $SiO_2$ shell was coated on csUCNPs to form YSUCNP. PdPc was loaded in YSUCNP to prepare YSUCNP-PdPc. DPPC and DOX were coated on YSUCNP-PdPc to prepare YSUCNP-PdPc@DPPC-DOX (TR-UCNS).

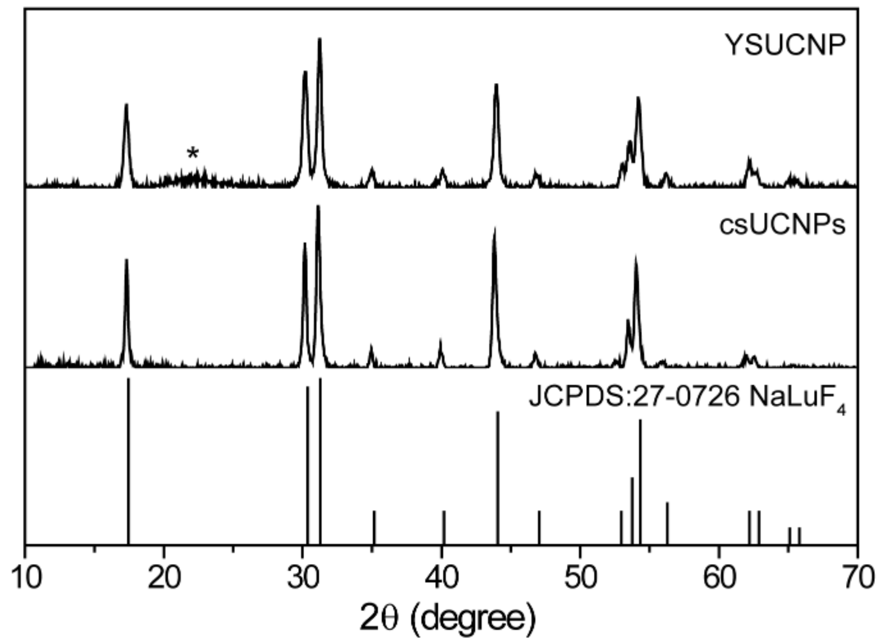


**Supplementary Figure 2.** XRD patterns of csUCNPs and YSUCNP. The standard pattern of pure hexagonal $NaLuF_4$ (JCPDS card No. 27-0725). The amorphous phase of $SiO_2$ component in YSUCNP is marked with an asterisk.

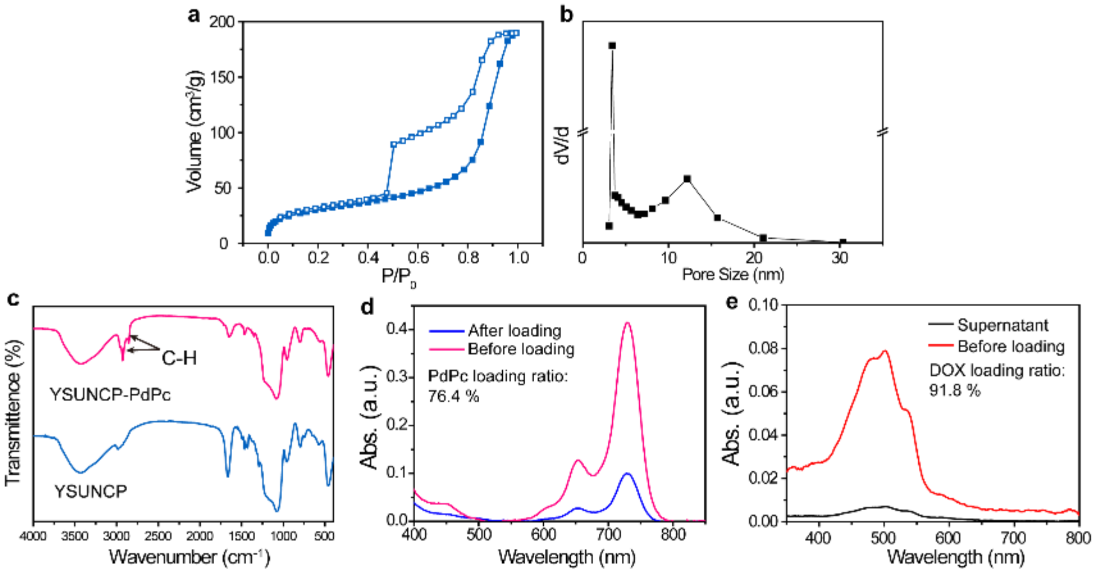


**Supplementary Figure 3. (a)** Nitrogen adsorption–desorption isotherms and **(b)** Pore size distribution curve of YSUCNP. The pore size distribution is derived from the adsorption branches of the isotherms based on Barrett–Joyner–Halenda (BJH) model. **(c)** Fourier transform infrared **(**FTIR) spectra of YSUCNP and YSUCNP-PdPc. The stretching bands referring to C-H in PdPc are marked with arrows. **(d)** Absorption spectra of PdPc dissolved in dichloromethane before and after loading on YSUCNP. The loading ratio of PdPc in YSUCNP is calculated to be 76.4 %. **(e)** Absorption spectra of DOX dissolved in ethanol before loading on YSUCNP@PdPc and supernatant after loading. The loading ratio of DOX in TR-UCNS is calculated to be 91.8 %.

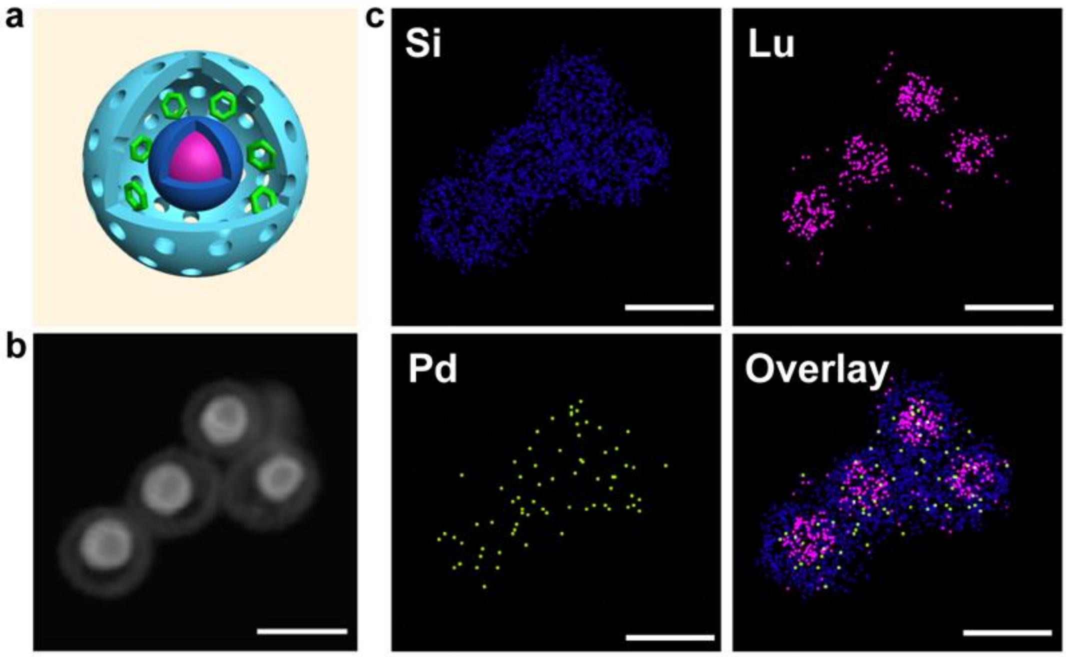


**Supplementary Figure 4. (a)** Schematic illustration of YSUCNP-PdPc. **(b)** High-angle annular dark-field (HAADF) images of YSUCNP-PdPc. Scale bar was defined as 50 nm. **(c)** Energy dispersive X-ray (EDX) analysis of YSUCNP-PdPc. In the elemental mapping images of YSUCNP-PdPc, the yolk-shell structure of YSUCNP can be clearly distinguished and the successful loading of PdPc in YSUCNP is also confirmed according to the existence of signals Pd element in the nanoparticles. Scale bars were defined as 50 nm.

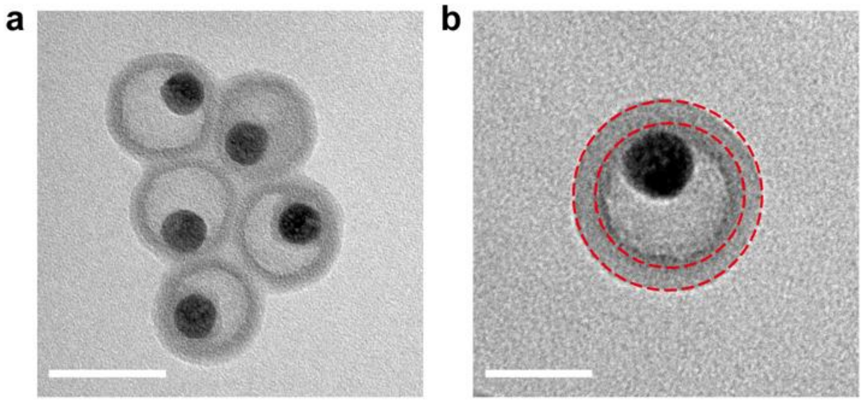


**Supplementary Figure 5. (a)** TEM image of YSUCNP-PdPc@DPPC-DOX. Scale bar was defined as 50 nm. **(b)** Magnified TEM image of single YSUCNP-PdPc@DPPC-DOX nanoparticle. The silica shell and DPPC layer are marked with red dash circles. Scale bar was defined as 30 nm.

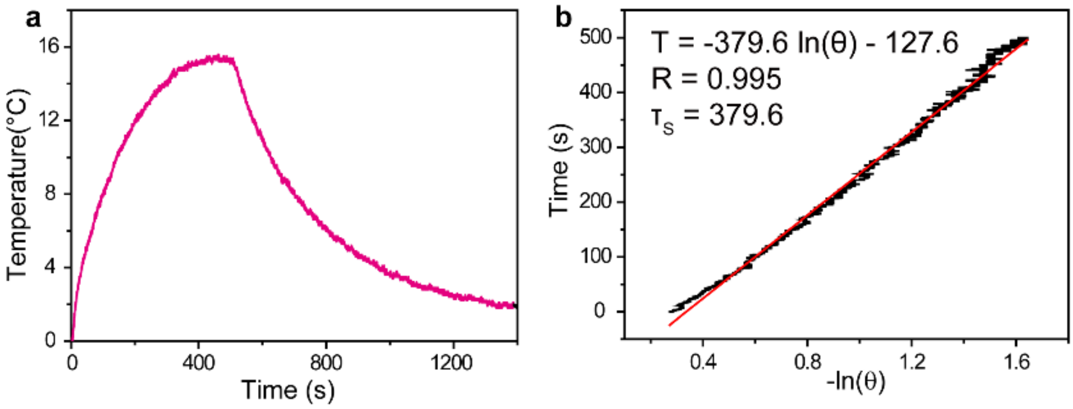


**Supplementary Figure 6. (a)** Photothermal effect of the irradiation of the aqueous dispersion of YSUCNP-PdPc@DPPC-DOX with the 730 nm laser (300 mW cm$^{-2}$). **(b)** Time constant for heat transfer from the system is determined to be $\tau_s$ = 379.6 s by applying the linear time data from the cooling period versus negative natural logarithm of driving force temperature, which is obtained from the cooling stage in **(a)**.

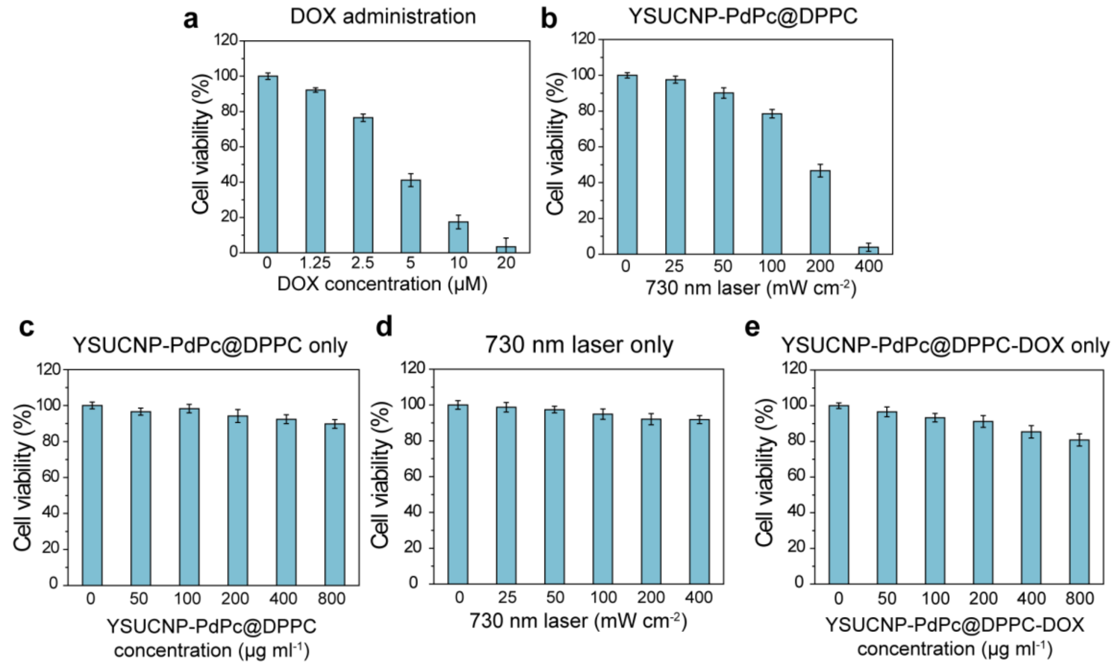


**Supplementary Figure 7.** Methyl thiazolyl tetrazolium (MTT) assays of MIA PaCa-2 cells treated with **(a)** DOX at different concentrations (0, 1.25, 2.5, 5, 10 or 20 μM), **(b)** YSUCNP-PdPc@DPPC at 200 μg $ml^{-1}$ under 730 nm laser irradiation for 5 min at 0, 25, 50,100, 200 and 400 mW $cm^{-2}$, **(c)** YSUCNP-PdPc@DPPC at different concentrations (0, 50, 100, 200, 400 or 800 μg $ml^{-1}$), **(d)** no nanomaterials under 730 nm laser irradiation for 5 min at 0, 25, 50,100, 200 and 400 mW $cm^{-2}$, **(e)** YSUCNP-PdPc@DPPC-DOX at different concentrations (0, 50, 100, 200, 400 or 800 μg $ml^{-1}$). All error bars in **a**-**e** were defined as s.d. ($n$ =3).

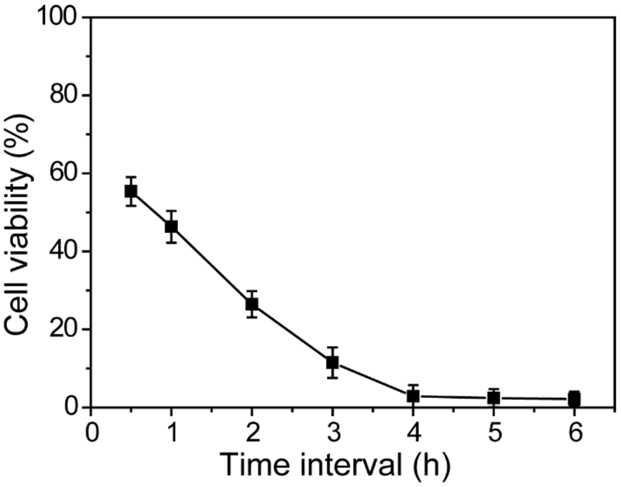


**Supplementary Figure 8.** Viability of MIA PaCa-2 cells treated with programmed combination therapy with different time intervals between Command 1 (Drug release) and Command 2 (Photothermal therapy). The power density of 730 nm laser for drug release and photothermal therapy are 46 mW $cm^{-2}$ and 140 mW $cm^{-2}$, respectively. Four hours' interval between Command 1 and 2 resulted in the maximized therapeutic effect so that this time interval was employed for in vitro and in vivo programmed combination therapy study. Error bars were defined as s.d. (*n* =3).

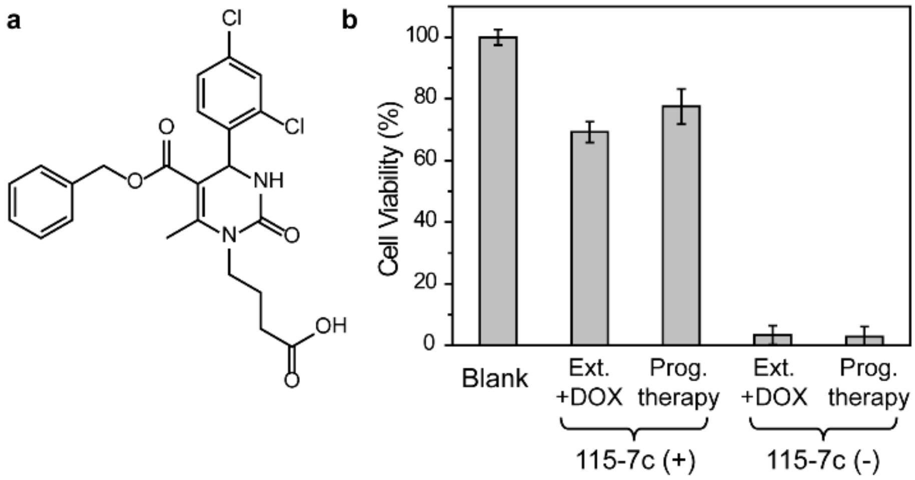


**Supplementary Figure 9. (a)** Molecular structure of 115-7c. **(b)** MTT assays of MIA PaCa -2 cells under two different conditions (Ext.+DOX: external heating to simulate the photothermal process and DOX or Prog. therapy: programmed combination therapy) with or without the incubation of 115-7c. Error bars were defined as s.d. (*n* =3).

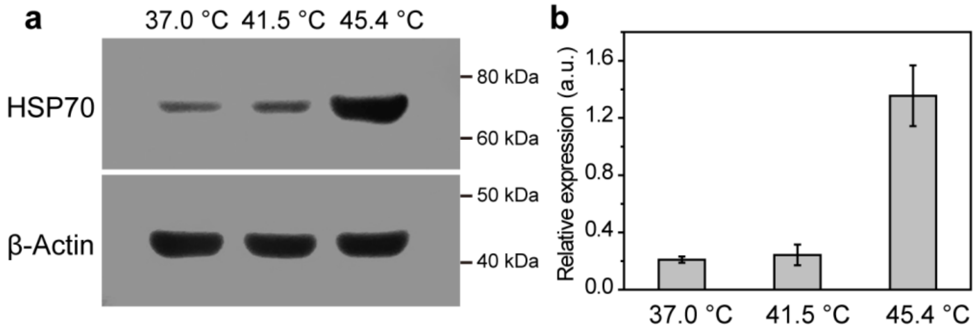


**Supplementary Figure 10. (a)** HSP70 expressions of MIA PaCa-2 cells under 37.0, 41.5 and 45.4 °C treatment with western blot analysis. β-Actin was used as internal control. **(b)** Quantitative data of the expression of HSP70 in MIA PaCa-2 cells under 37.0, 41.5 and 45.4 °C treatment. Error bars were defined as s.d. ($n$ =3).

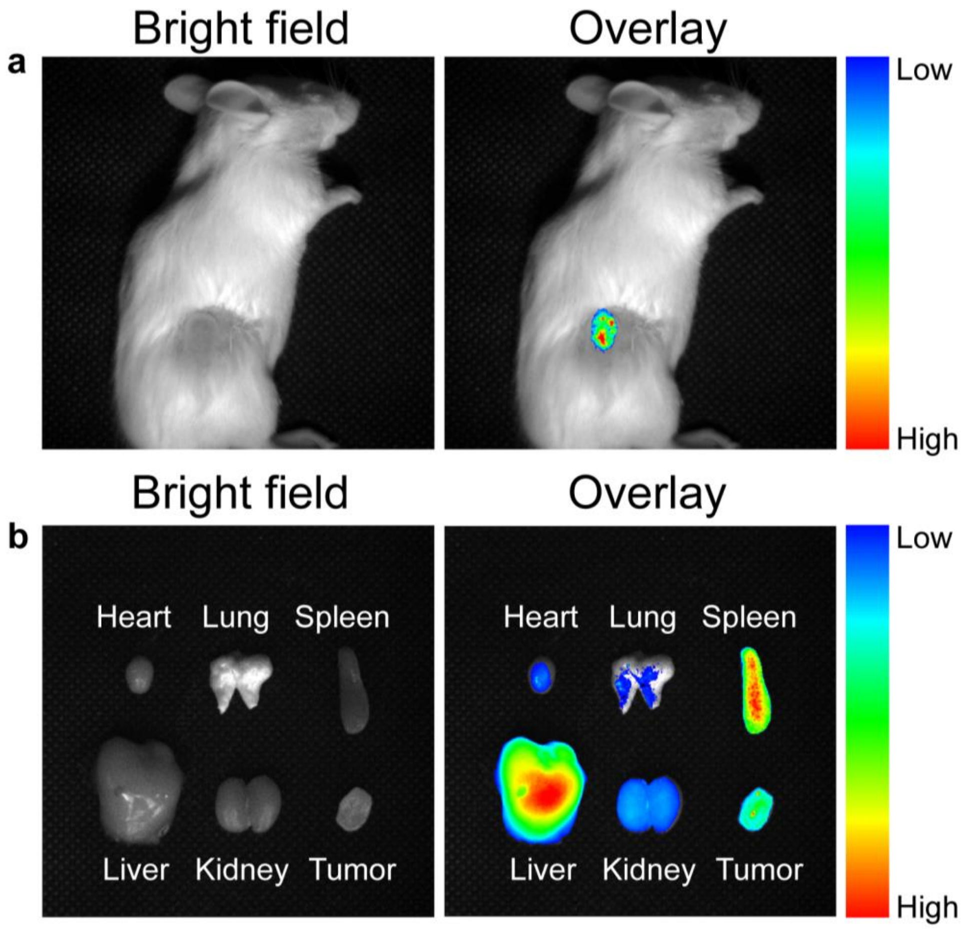


**Supplementary Figure 11.** Tumor targeted upconversion luminescence imaging. **(a)** *In vivo* UCL imaging of tumor-bearing mice 6 h after intravenous injection of TR-UCNS (2 mg ml$^{-1}$, 200 μl). Left, bright field image of tumor bearing Balb/c scid mice. Right, overlay of bright field and UCL images. **(b)** *Ex vivo* UCL image of the major organs of the injected mice (heart, lung, spleen, liver, kidney and tumor).

Left, bright field image of tumor bearing Balb/c scid mice. Right, overlay of bright field and UCL images.

UCL signals were collected by using a 720 nm short pass filter.

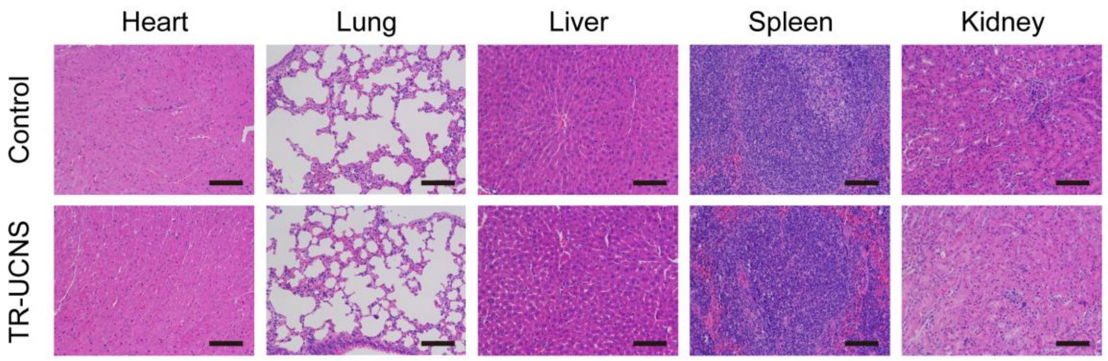


**Supplementary Figure 12.** Histological changes in the heart, lung, liver, spleen, kidney of mice one week after intravenous injection of YSUCNP-PdPc@DPPC-DOX (200 µl, 2 mg $ml^{-1}$). The organs are stained with hematoxylin and eosin (H&E). Scale bars were defined as 100 µm.

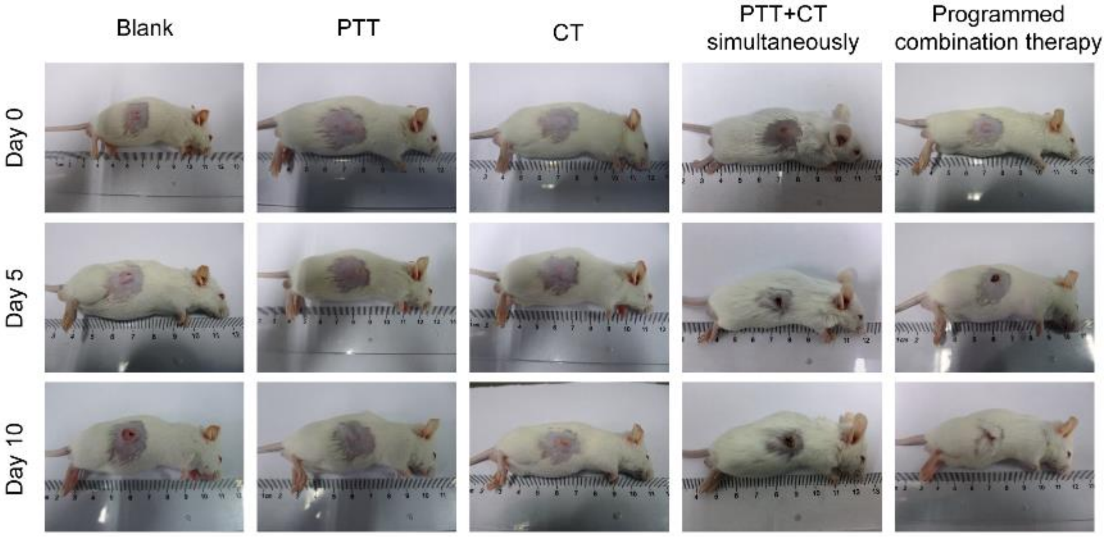


**Supplementary Figure 13.** Representative photos of tumor-bearing Balb/c scid mice before and after treatment. The tumors in the mice injected with YSUCNP-PdPc@DPPC-DOX (200 μl, 2 mg $ml^{-1}$) with programmed combination therapy shrank, while the other groups (blank, photothermal therapy or chemotherapy alone, and photothermal therapy and chemotherapy performed simultaneously) showed a weak suppressing effect on tumor growth.

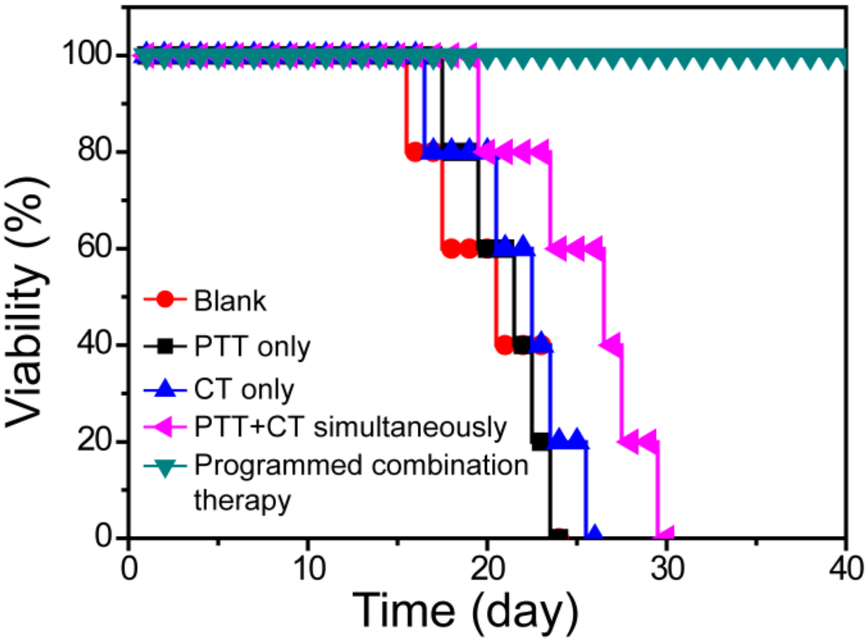


**Supplementary Figure 14.** Survival curves of mice after various treatments.

**Supplementary Methods**

**Materials and Characterization** All the starting materials were obtained from commercial supplies and used as received. All chemicals were used as received without further purification. Lanthanide oxides $Lu_2O_3$ (99.999%), $Yb_2O_3$ (99.999%) and $Er_2O_3$ (99.999%) were purchased from Shanghai Yuelong New Materials Co. Ltd. China. Oleic acid (OA) (>90%), 1-octadecence (ODE) (>90%), 1,4,8,11,15,18,22,25-octabutoxy-29H,31H-phthalocyanine (95%), doxorubicin hydrochloride (DOX·HCl), Igepal CO-520, 1,2-Dipalmitoyl-sn-glycero-3-phosphocholine (DPPC) (≥99%), palladium(II) chloride and N-[3-(trimethoxysilyl)propyl]ethylenediamine (TSD) (97%) was purchased from Sigma–Aldrich Co., Ltd. NaOH, $NH_4F$, $CHCl_3$, hydrofluoric acid, methanol, ethanol, cyclohexane, hydrochloric solution, tetraethyl orthosilicate (TEOS), dimethylformamide (DMF) were purchased from Sinopharm Chemical Reagent Co., China. Rare-earth chlorides ($LnCl_3$, Ln: Lu, Yb and Er) were prepared by dissolving the corresponding metal oxide in 10% hydrochloric solution at elevated temperature and then evaporating the water completely. Deionized water was used throughout the experiments.

Powder X-ray diffraction (XRD) measurements were performed by a Bruker D4 X-ray diffractometer (Cu radiation, 0.15406 nm). The morphologies of nanoparticles were observed at 200 kV on a JEOL JEM-2010F low- to high-resolution transmission electron microscope (HRTEM). Samples were prepared by placing a drop of dilute dispersions in cyclohexane and water on the surface of a copper grid respectively. Energy-dispersive X-ray (EDX) elemental mapping of the samples was also performed during HRTEM measurements to obtain the elements of samples. UV/Vis spectra were obtained with a UV-Vis Spectrophotometer (Shimadzu 3000). Nitrogen sorption isotherms were measured at 77 K with a Micromeritics Tristar 3000 analyzer. Upconversion luminescence (UCL) spectra were measured on an Edinburgh LFS-920 spectrometer, where an external 0-3 W adjustable CW laser at 980 nm (Connet Fiber Optics, China) replaced the Xenon lamp as the excitation source. Confocal luminescence imaging of tissues of mouse was performed with on a modified OLYMPUS FV1000 laser scanning confocal fluorescence microscope equipped with a continuous-wave NIR laser operating at 980 nm (Connet Fiber Optics, China).

**Calculation of the photothermal conversion efficiency.**

According to the method described in the literature, the total energy conservation for the system can be expressed by Supplementary Equation 1.

$$\sum_i m_i C_{p,i} \frac{dT}{dt} = Q_{cs} + Q_B - Q_{sur} \quad (1)$$

where $m$ and $C_p$ are the mass and heat capacity of water respectively, $T$ is the solution temperature, $Q_{cs}$ is the energy induced by TR-UCNS, $Q_B$ is the baseline energy induced by the sample cell, and $Q_{sur}$ is heat conduction away from the surface by air.

$Q_{cs}$ is caused by the π-plasmon of the carbon shell under irradiation of 730 nm laser:

$$Q_{cs} = I(1 - 10^{-A_{730}})\eta \quad (2)$$

where $I$ is the laser power, $\eta$ is the conversion efficiency from incident laser energy to thermal energy, and $A_{730}$ is the absorbance of TR-UCNS at wavelength of 730 nm. On the other hand, $Q_B$, expressing heat dissipated from light absorbed by the sample cell, was measured independently to be 26.8 mW using a quartz cuvette containing pure water without TR-UCNS. Moreover, $Q_{sur}$ is in proportion to temperature for the outgoing thermal energy, as given by Supplementary Equation 3.

$$Q_{sur} = hS(T - T_{\mathrm{amb}}) \quad (3)$$

where $h$ is heat transfer coefficient, $S$ is the surface area of the container, and $T_{amb}$ is ambient temperature of the surroundings.

According to Supplementary Equation 3, when the system temperature will reach a maximum, the heat input is equal to heat output:

$$Q_{CS} + Q_B = hS(T_{max} - T_{amb}) \quad (4)$$

where $T_{max}$ is the equilibrium temperature. The 730 nm laser heat conversion efficiency ($\eta$) can be determined by substituting Supplementary Equation 2 for $Q_{CS}$ into Supplementary Equation 4 and rearranging to get

$$\eta = \frac{hS(T_{max} - T_{amb}) - Q_B}{I(1 - 10^{-A_{730}})} \quad (5)$$

where $Q_B$ was measured independently to be 22.7 mW, the ($T_{max}$-$T_{amb}$) was 15.6 °C according to Supplementary Figure 6, $I$ is 300 mW cm$^{-2}$, $A_{730}$ is the absorbance (1.126) of TR-UCNS at 730 nm (Figure 3b). Here, $hS$ is calculated by introducing $\theta$, is defined as the expression below:

$$\theta = \frac{T - T_{amb}}{T_{max} - T_{amb}} \quad (6)$$

and a sample system time constant $\tau_s$

$$\tau_s = \frac{\sum_i m_i C_{p,i}}{hs} \quad (7)$$

which is substituted into Supplementary Equation 4 and rearranged to yield

$$\frac{d\theta}{dt} = \frac{1}{\tau_s}\left[\frac{Q_{cs}+Q_B}{hS(T_{max}-T_{amb})} - \theta\right] \quad (8)$$

At the cooling stage of the aqueous dispersion of the TR-UCNS, the light source was shut off, the $Q_{CS} + Q_B = 0$, reducing the Supplementary Equation 9

$$dt = -\tau_s \frac{d\theta}{\theta} \quad (9)$$

and integrating, giving the expression

$$t = -\tau_s \ln\theta \quad (10)$$

Therefore, time constant for heat transfer from the system is determined to be $\tau_s$ = 379.6 s by applying the linear time data from the cooling period (after 520 s) *vs* negative natural logarithm of $\theta$ (Supplementary Figure 6). In addition, the $m$ is 1 g and the $C$ is 4.2 J $g^{-1}$. Thus, according to Supplementary Equation 7, the $hS$ is deduced to be 11.1 mW $°C^{-1}$. Substituting 11.1 mW $°C^{-1}$ into the $hS$ into Supplementary Equation 5, the 730 nm laser heat conversion efficiency ($\eta$) of TR-UCNS can be calculated to be 54.2 %.

**Chemical induction of heat shock protein 70 (HSP70).** HSP70 activator, 115-7c (StressMarq Inc.), was used to induce the generation of HSP70 in MIA PaCa-2 cells. Cells were incubated with 10 μM 115-7c for 6 h and then cells were treated with programmed combination therapy or doxorubicin incubation (2.5 μM) for 4 h followed by external heating at 45.4 °C for 5 min. Cells without 115-7c incubation were also treated with programmed combination therapy or doxorubicin incubation (2.5 μM) for 4 h followed by external heating at 45.4 °C for 5 min as control. MTT assay were used to evaluate the cell viability.

**Synthesis of tissue phantom[1].** 3 ml Tris-buffered saline containing TR-UCNS (0.5 mg $ml^{-1}$) was mixed with a certain amount of sodium azide and gelatin to a final concentration of 15 mM and 10 w/v%, respectively. Then the solution was heated to 50°C under constant stirring. When the gelatin was totally dissolved, the mixture was kept at 37°C. Hemoglobin and intralipid were added to the system to a concentration of 50 μM and 1 v/v%. Finally, the mixed solution was poured into a quartz cuvette for subsequent UCL spectra acquisition. As reported in the literature, the absorption coefficient ($m_a$) and the scattering coefficient ($m_s$) at 700 nm were approximately 1.0 $mm^{-1}$ and 0.5 $mm^{-1}$, respectively[1]. This gelatin-based phantom mimicked physiological tissue and represented a

good physiological model for *in vivo* tissue.

**Histological analysis.** One week after injection of TR-UCNS, mice were sacrificed and tumor tissues and major organs from these mice were harvested, fixed in 5% polyoxymethylene solution, routinely processed in paraffin, sectioned at 8 μm, stained with hematoxylin and eosin (H&E), and observed using a digital microscope. Examined tissues included liver, spleen, kidney, heart, lung and tumor.

## Supplementary References

1. De Grand, A.M., *et al.* Tissue-like phantoms for near-infrared fluorescence imaging system assessment and the training of surgeons. *J. Biomed. Opt.* **11**, 014007 (2006).
